# Clonidine Overdose as an Unusual Cause of Heart Failure

**DOI:** 10.1177/23247096221106856

**Published:** 2022-06-24

**Authors:** Jagdeep Bhullar, Arti Patel, Jaagruthi Chitithoti, Frederick Venter, Theingi Win, Fowrooz Joolhar

**Affiliations:** 1 Kern Medical Center, Bakersfield, CA, USA; 2 University of California, San Francisco, Fresno, USA

**Keywords:** heart failure, clonidine overdose, HFrEF, troponemia, NSTEMI, GDMT, cardiology

## Abstract

Clonidine is used as an antihypertensive medication due to its effect on decreasing peripheral vascular resistance and therefore lowering blood pressure. Alpha antagonism in the medulla and the posterior hypothalamus causing a reduction in sympathetic activation allows for clonidine to be used as an effective off-label treatment for attention-deficit/hyperactivity disorder (ADHD). This is a case of a 28-year-old female with hypertension, ADHD, and depression who developed acute heart failure with significant troponemia after ingesting 30 pills of clonidine. We illustrate the possible rare diagnosis of systolic heart failure and coronary vasospasm secondary to clonidine overdose.

## Introduction

Clonidine overdose most commonly presents with hypotension, bradycardia, and altered mental status.^1^ Clonidine’s alpha-agonist properties decrease stimulation of receptors in the heart, vessels, and kidneys resulting in decreased vascular resistance, heart rate, and cardiac contractility while increase vasodilation.^2,3^ However, here we discuss the management of a suicidal patient diagnosed with systolic heart failure and coronary vasospasm secondary to clonidine overdose.

## Case Presentation

**Figure 1.**
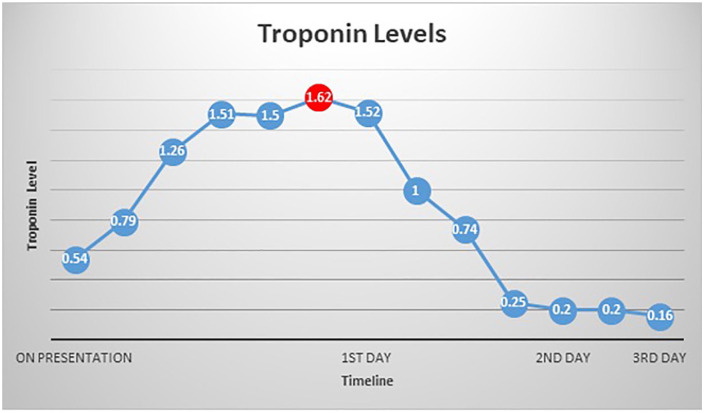
Line graph of troponin levels of the patient. Troponin levels ranged between 0.54 and 1.52 on the first day, with a peak of 1.62 highlighted in the red dot. Troponin levels normalized to 0.20.

**Figure 2.**
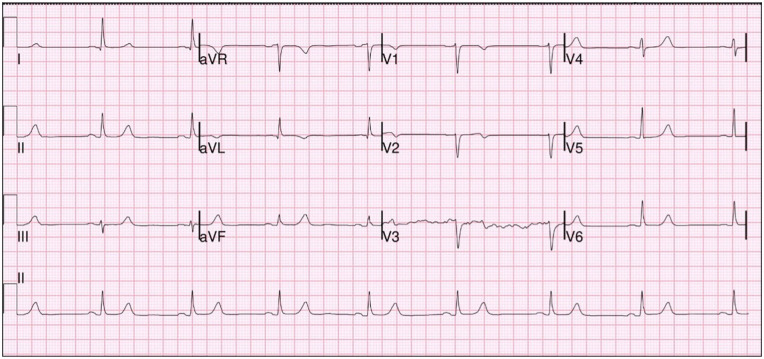
Patient EKG (electrocardiogram) at admission showing Q waves.

**Figure 3.**
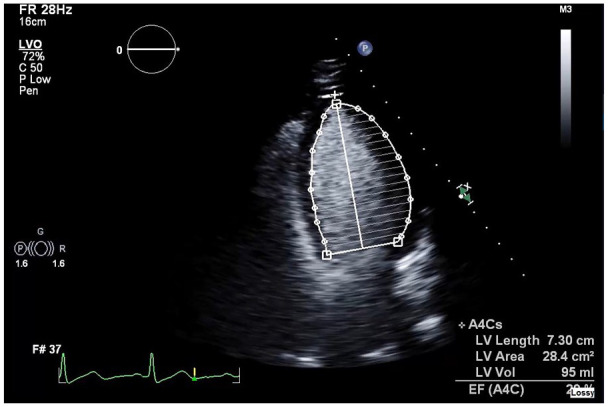
Echocardiogram on admission revealing dilation of the left ventricle.

A 28-year-old female with hypertension, attention-deficit/hyperactivity disorder (ADHD), and depression presented to the emergency department (ED) altered after being found down by family after ingesting 30 pills of clonidine. She reported 5 days of cold-like symptoms including subjective fevers, sore throat, body aches, and 3 days of sharp, constant, bilateral chest pain prior to her suicide attempt. After returning to her baseline mentation, the patient denied any current cardiac symptoms including chest pain, shortness of breath, dyspnea on exertion, orthopnea, paroxysmal nocturnal dyspnea, edema, palpitations, lightheadedness, or syncope along with no history of cardiac complications. Vitals showed bradycardia with heart rate in the 50 to 60s, but normotensive and afebrile. Lab values showed significant troponemia which ranged from 0.54 to 1.52 and peaked at 1.62 (Figure 1). EKG (electrocardiogram) showed Q waves concerning for STEMI (ST-elevation myocardial infarction) (Figure 2) and the patient was admitted for STEMI and ischemic workup. ACS (acute coronary syndrome) protocol was initiated and then discontinued after downtrending troponins and resolution of cardiac symptoms. Echocardiogram showed dilated cardiomyopathy (Figure 3) with severe left ventricular systolic dysfunction (LVEF) of 25% to 30% and concentric left ventricular hypertrophy. STEMI was ruled out as the angiogram revealed no coronary artery disease with normal coronaries. Troponemia spontaneously resolved and the patient was optimized on GDMT (guideline-directed medical therapy) and is doing well. The most recent echocardiogram revealed an LVEF of 50%.

## Discussion

Clonidine is used as an antihypertensive medication due to its effect on decreasing peripheral vascular resistance and therefore lowering blood pressure. It is also an effective off-label treatment for ADHD, such as in this case.^4^ During a suicide attempt, the patient ingested 30 pills of clonidine and presented to the ED with somnolence, chest pain, and bradycardia. Clonidine overdose usually presents as altered mental status, hypotension, and bradycardia.^1^ Many case reports have documented the occurrence of somnolence and weakness after acute clonidine intoxication.^5,6^ Cases of clonidine-induced paradoxical hypertension have also been documented.^7^ However, there have been very few documented cases of clonidine overdose causing an NSTEMI (non-ST-elevation myocardial infarction) with chest pain, EKG changes, and elevated troponin. Subsequent evaluation with echocardiogram and angiogram revealed no coronary artery disease, which suggests that her NSTEMI presentation came most likely from clonidine-induced vasospasm. Echocardiogram showing dilated cardiomyopathy and severe LVEF dysfunction suggests clonidine-induced heart failure as seen in this case. Similarly, in another case study, a patient experienced hypertensive urgency, seizure, and myocardial infarction after a large dose of parenteral clonidine.^8^ The pathophysiology of clonidine-induced infarction stems from how clonidine at doses higher than 7 mg/d can cause vasoconstriction by acting on peripheral alpha 1 and alpha 2 adrenergic receptors.^8^ An overstimulation of the peripheral alpha receptors secondary to clonidine overdose has been shown to cause vasospasm and paradoxical hypertension, as exemplified in our case study.^8^ As an alpha-adrenergic receptor agonist, clonidine can activate both central alpha 2 adrenergic receptors and peripheral alpha 1 and alpha 2 adrenergic receptors.^9^ Activation of central alpha 2 receptors causes an inhibitory reaction on sympathetic output, therefore, causing a reduction of norepinephrine secretion which in turn reduces blood pressure and heart rate.^9^ Our patient’s bradycardia on presentation was likely due to clonidine’s central alpha 2 activation. Paradoxically, activation of peripheral alpha 1 receptors causes vasoconstriction.^9^ Furthermore, activation of peripheral alpha 2 receptors causes increased catecholamine release leading to paradoxical hypertension.^9^ Although hypotension and bradycardia are the most common symptoms in alpha-agonist overdose, high doses of alpha-agonist ingestion can cause paradoxical peripheral vasoconstriction, such as seen in our patient.^9,10^ In clonidine overdose, transient toxicity of peripheral alpha 1 receptors and post-synaptic alpha 2 receptors in cardiac vascular smooth muscle causes transient hypertension along with peripheral vasoconstriction.^9,11^ Due to clonidine’s peripheral alpha effects, alpha 1 and alpha 2 receptors are activated in coronary vascular smooth muscle, epicardial coronary arteries, and coronary resistance vessels, along with collaterals, possibly causing vasospasm in various coronary vessels.^12^ Due to this peripheral alpha activation causing an increase in norepinephrine-induced vasoconstriction, the clonidine overdose manifested as an NSTEMI presentation in our patient’s case. This clonidine-induced vasospasm could have also been the cause of the patient’s severe LVEF dysfunction of 25% to 30% noted in her echocardiogram. Clonidine-induced vasospasm is another unique presentation of clonidine overdose that has been discussed through this case.

## Conclusion

Consider clonidine-induced vasospasm as a differential diagnosis in NSTEMI patients with clonidine overdose. Although the most common presentation of clonidine overdose is altered mental status, hypotension, and bradycardia, a patient may present with chest pain, EKG changes, and elevated troponin.^1^ Although patients may be young with no risk factors for heart failure or NSTEMI, patients may have experienced coronary vasospasm secondary to the clonidine overdose. Treat these patients with ACS protocol, however, continue low-dose beta-blocker and angiotensin-converting enzyme inhibitor. Diagnosis can be verified with an angiogram. Repeat echocardiogram can monitor the resolution of acute heart failure after discharge. A multidisciplinary approach with cardiology is recommended.
